# Post-operative procalcitonin and C-reactive protein predict pancreatic fistula after laparoscopic pancreatoduodenectomy

**DOI:** 10.1186/s12893-021-01177-4

**Published:** 2021-03-30

**Authors:** Jie Ma, Peiqiang Jiang, Bai Ji, Yanqing Song, Yahui Liu

**Affiliations:** 1 Department of Pharmacy, First Hospital of Jilin University, No. 1 Xinmin Road, Changchun, 130021 Jilin China; 2 Department of Hepatobiliary and Pancreatic Surgery, First Hospital of Jilin University, No. 1 Xinmin Road, Changchun, 130021 Jilin China

**Keywords:** Laparoscopic pancreaticoduodenectomy, Pancreatic fistula, Procalcitonin, C-reactive protein

## Abstract

**Background:**

Clinically relevant pancreatic fistula (CRPF) is a serious complication following laparoscopic pancreaticoduodenectomy (LPD). This study aimed to determine if C-reactive protein (CRP) and procalcitonin (PCT) serum levels could be used as early biomarkers to predict CRPF after LPD.

**Methods:**

In this retrospective study, we collected peri-operative data of patients who underwent LPD between January 2019 and November 2019. We compared serum levels of white blood cells (WBC), CRP, and PCT on post-operative days (POD) 1, 2, 3, 5, and 7 between the CRPF and non-CRPF groups and analyzed the predictive risk factors for CRPF.

**Results:**

Among the 186 patients included in this study, 18 patients (9.7%) developed CRPF, including 15 and 3 patients with grade B and C fistulas, respectively. The mean WBC, CRP, and PCT levels were higher on most PODs in the CRPF group compared to the non-CRPF group. Receiver operating characteristic (ROC) analysis indicated that CRP levels on POD 2, 5, and 7 can predict CRPF development after LPD, with the area under the curve (AUC) value reaching the highest level on POD 2 (AUC 0.794). PCT levels on POD 2, 3, 5, and 7 were highly predictive of CRPF after LPD. The highest AUC value was achieved on POD 3 [PCT > 2.10 ng/ml (AUC 0.951; sensitivity 88.2%, specificity 92.9%, *P* < 0.001)].

**Conclusions:**

Both CRP and PCT levels can be used to predict CRPF development after LPD, with PCT having a higher predictive value.

## Background

Laparoscopic pancreaticoduodenectomy (LPD) was first described in 1994 [1]. Since then, LPD has been increasingly performed in high volume pancreatic centers worldwide. LPD is a challenging procedure, requires advanced laparoscopic skills, and is associated with a long learning curve [2]. Post-operative pancreatic fistula (POPF) is a serious complication after LPD due to the risk of secondary bleeding and intra-abdominal infections [3]. The occurrence of a POPF prolongs post-operative hospital stays and causes an increased risk of mortality. Most comparative studies of LPD and open PD (OPD) have found no significant difference in the incidence of POPF [4, 5]. However, some studies have reported a higher incidence of POPF following LPD [6, 7]. According to recent studies, the incidence of clinically relevant pancreatic fistula (CRPF) after LPD, as defined by the 2016 International Study Group of Pancreas Surgery, ranges between 6.5 and 10.8% [2, 8, 9]. Accurate prediction and timely diagnosis of CRPF after LPD is crucial for improving patient management, providing timely treatment (such as percutaneous, endoscopic, or surgical drainage), reducing hospital stay, and preventing mortality. Several studies have used radiological imaging [10], laboratory parameters [11], and clinical scoring systems [12] to predict POPF development after OPD.

White blood cell (WBC) counts, C-reactive protein (CRP), and procalcitonin (PCT) are the most commonly used markers of inflammation. Elevated blood levels of these markers are associated with inflammatory or infectious conditions. CRP and PCT have been widely used as early predictors of anastomotic leak and infection in colorectal surgery [13], gastrointestinal surgery [14], and pancreatic surgery [15]. Prior reports have demonstrated that CRP and PCT are accurate predictors of infective complications after OPD [16–19]. However, LPD is a more complex operation and requires highly skilled surgeons. Most hospitals do not perform LPD, and only some hospitals perform a few LPD operations every year, making it difficult to conduct any meaningful analysis from patient data. Furthermore, there are no published studies that report levels of inflammatory markers after LPD, let alone early predictors of CRPF. In this study, we aim to describe the kinetics of CRP and PCT after LPD and compare their usefulness for early detection of CRPF after LPD using data from our medical center.

## Methods

### Study design and patients

This was a retrospective analysis of data obtained from electronic medical records. Patients who underwent LPD between January 2019 and November 2019 in the Department of Hepatobiliary and Pancreatic Surgery at the First Hospital of Jilin University (Changchun, China), a large tertiary grade A hospital and high-volume pancreatic surgical center, were included in this study. The study protocol was approved and informed consent was waived due to the retrospective nature of this study by the Ethical Committee of the First Hospital of Jilin University (Ethics Approval Number: 2019–232).

All operations were performed by the same senior pancreatic surgeon with the help of a dedicated staff. There was no bias in the selection of patients. Exclusion criteria were as follows: (i) patients with symptoms and signs of active infection at the time of surgery, (ii) patients with autoimmune disorders or hematological malignancies, and (iii) incomplete clinical data.

### LPD procedure

The patients were placed in a supine position with legs separated. A 12 mm camera port was inserted 3 cm below the umbilicus and pneumoperitoneum was established. The intra-abdominal pressure was maintained at 12–14 mmHg. The surgeon stood on the right side of the patient, the assistant stood on the left side, and the laparoscope holder stood between the legs of the patient. The operation was performed using the posterior colonic approach. The pancreatico-jejunal, bilio-enteric, and gastrojejunal anastomoses were performed laparoscopically as described previously [20]. Reconstruction was performed using the same technique in all the patients.

### Post-operative care

All patients were transferred to the intensive care unit (ICU) for monitoring after surgery. Patients were transferred to general wards within 1–2 days once all vital parameters stabilized. All patients received prophylactic antibiotic treatment. According to the fast-track protocol, all patients had their nasogastric tubes removed on the first day after surgery, and liquid food was administered once flatus was passed. The drainage volume was closely observed after surgery. If the drainage volume was < 50 mL/d and the amylase level in the ascitic fluid was < 3000 IU/L, the abdominal drain was removed on the third day after surgery.

The WBC count, CRP, and PCT levels were routinely measured on post-operative days (PODs) 1, 2, 3, 5, and 7 after LPD. We also collected data from pre-operative laboratory testing, intra-operative events, and post-operative outcomes.

POPF was defined in this study using the International Study Group of Pancreatic Surgery (ISGPS) 2016 criteria [21] and classified into three types: biochemical leak, grade B fistula, and grade C fistula. In the present study, grade B and C fistulas were defined as CRPF.

### Statistical analysis

Continuous variables with non-normal distribution are represented as median (Q1–Q3) and compared using the Mann–Whitney U test. Categorical variables are represented as frequency (percentage) and compared using the Chi-square or Fisher’s exact test. Multivariate analysis was carried out using logistic regression analysis. The best cutoff points for the predictive variables of CRPF were identified using receiver operating characteristic (ROC) curves and the Youden index. The area under the curve (AUC) of the different ROC curves were compared using Delong’s test [22]. All analyses were conducted with SPSS (version 18.0, Chicago, IL, USA), and *P* values < 0.05 were considered statistically significant.

## Results

### Baseline characteristics and post-operative course

A total of 194 patients underwent LPD during the study period. Among these patients, 8 patients were excluded due to presence of active infection (n = 3), presence of autoimmune disorder (n = 1), presence of hematological malignancy (n = 1), and incomplete clinical data (n = 3). Finally, 186 patients were included in this study. There were 102 males and 84 females with a median age of 61 years (interquartile range (IQR), 52–67). No patient required conversion to open surgery. CRPF developed in 18 patients (grade B fistula in 15 patients and grade C fistula in 3 patients). The incidence of CRPF was 9.7%. Table 1 summarizes the patients’ clinical characteristics, pre-operative laboratory data, intra-operative events, and post-operative outcomes in the CRPF and non-CRPF groups. There were significant.

**Table 1:** Comparison of clinical characteristics, pre-operative laboratory parameters, intra-operative events, and post-operative outcomes between the CRPF and non-CRPF groups

Variables	CRPF group (n = 18)	non-CRPF group (n = 168)	*P* value
Age (years)	64 (60–69)	60 (52–67)	0.103
Gender (male/female)	7/11	95/73	0.152
BMI (kg/m^2^)	22.7 (21.5–25.3)	22.0 (20.2–23.4)	0.121
Diabetes	2 (11.1%)	19 (11.3%)	1.000
History of abdominal operation	3 (16.7%)	28 (16.7%)	1.000
Obstructive jaundice	7 (38.9%)	77 (45.8%)	0.574
Pre-operative biliary drainage	7 (38.8%)	53 (31.5%)	0.527
White blood cells (× 10^9^/L)	5.83 (5.37–7.59)	5.72 (4.75–6.84)	0.439
Alanine aminotransferase (U/L)	36.4 (13.5–150.4)	100.4 (35.4–218.0)	0.054
Aspartate aminotransferase (U/L)	23.2 (17.7–91.4)	63.3 (28.3–150.2)	0.064
Total bilirubin (μmol/L)	27.7 (14.3–133.9)	73.8 (19.4–138.9)	0.386
Direct bilirubin (μmol/L)	19.6 (3.1–92.4)	47.6 (6.0–98.2)	0.319
Serum albumin (g/L)	39.4 (34.3–41.7)	37.8 (34.4–41.3)	0.587
Hemoglobin (g/L)	125 (104.5–134.0)	126 (112.2–140.0)	0.465
Blood creatinine (μmol/L)	60.1 (47.3–68.6)	59.3 (49.4–68.1)	0.899
Blood urea nitrogen (mmol/L)	4.32 (3.18–6.11)	4.91 (3.86–5.97)	0.506
Operation time (min)	240 (190.0–295.0)	247.5 (220.0–290.0)	0.403
Blood loss (mL)	50 (27.5–50)	50 (30–50)	0.374
Blood transfusion (mL)	0 (0–400)	0 (0- 0)	0.461
Pathology (pancreatic adenocarcinoma /others)	3/15	44/124	0.569
Pancreatic gland texture (soft/firm)	16/2	98/70	0.011
Pancreatic duct diameter (< 3 mm/ ≥ 3 mm)	12/6	70/98	0.042
Pathologic type (benign/malignant)	4/14	31/137	0.751
Reoperation	3 (16.7%)	15 (8.9%)	0.390
Other major complications			
Biliary fistula	2 (5.6%)	8 (4.8%)	1.000
Post-operative hemorrhage	5 (27.8%)	9 (5.3%)	0.006
Delayed gastric emptying	2 (11.1%)	24 (14.3%)	1.000
Post-operative hospital stay (days)	18.0 (11.5–32.5)	12.0 (9.0–15.0)	0.010
Mortality (%)	2 (11.1%)	2 (1.19%)	0.047

Data are expressed as the median [IQR] and number (percentage). Continuous variables with non-normal distribution were compared using the Mann–Whitney U test. Differences in the values of categorical variables were compared using the Chi-square test or Fisher’s exact test

*CRPF* clinically relevant pancreatic fistula

differences between the two groups regarding pancreatic gland texture and pancreatic duct diameter. In addition, patients in the CRPF group had higher incidences of post-operative hemorrhage, longer hospital stays, and higher mortality rates.

### Comparison of trends in WBC, CRP, and PCT between two groups

**Fig. 1:**
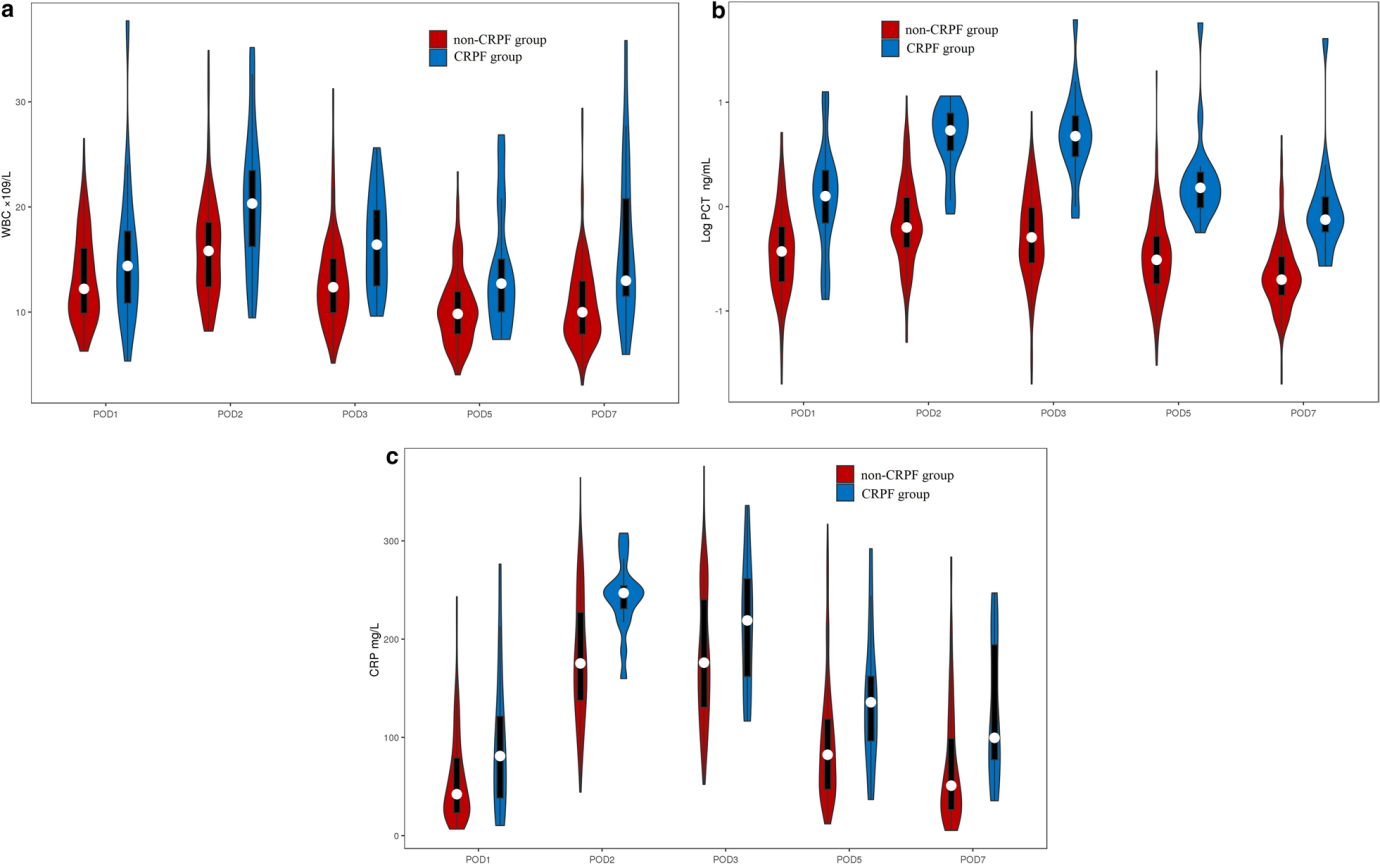
Grouped violin plots showing the distribution of WBC (× 10^9^/L) (A), PCT (ng/mL) (B), and CRP (mg/L) (C) in the CRPF and non-CRPF groups after LPD on PODs 1, 2, 3, 5, and 7. Considering the skewed distribution of post-operative PCT levels, we used the logarithmic variables. The shape of the distribution (extremely narrow on each end and wide in the middle) indicates that the inflammatory marker values were highly concentrated around the median. *CRP* C-reactive protein, *WBC* white blood cell, *PCT* procalcitonin, *POD* post-operative day, *CRPF* clinically relevant pancreatic fistula, *LPD* laparoscopic pancreaticoduodenectomy

Post-operative trends of WBC counts and PCT levels were similar in the CRPF and non-CRPF groups, with a peak on POD 2 (Fig. 1a and b). CRP levels peaked on POD 2 in the CRPF group and on POD 3 in the non-CRPF group (Fig. 1c). WBC counts on PODs 2, 3, 5, and 7, PCT levels on PODs 1, 2, 3, 5, and 7, and CRP levels on PODs 1, 2, 5, and 7 were significantly higher in the CRPF group (*P* < 0.001). Drain fluid amylase (DFA) on POD 3 was also significantly higher in the CRPF group (*P* < 0.001) (Table 2).

**Table 2:** Post-operative WBC, CRP, and PCT levels stratified by CRPF

	CRPF group (n = 18) Median (IQR)	Non-CRPF group (n = 168) Median (IQR)	*P value*
WBC (× 10^9^/L)			
POD1	13.82 (10.72–15.52)	12.50 (9.94–16.28)	0.242
POD2	19.97 (15.93–23.36)	16.30 (12.47–18.80)	0.003
POD3	15.54 (12.76–20.01)	12.52 (9.91–15.78)	0.001
POD5	13.08 (10.02–20.82)	9.85 (7.72–11.97)	0.009
POD7	15.35 (10.91–20.79)	9.66 (7.86–13.24)	< 0.001
PCT (ng/mL)			
POD1	1.33 (0.70–2.34)	0.31 (0.18–0.64)	< 0.001
POD2	5.76 (3.92–8.21)	0.63 (0.40–1.26)	< 0.001
POD3	3.88 (2.67–6.02)	0.51 (0.29–1.00)	< 0.001
POD5	1.51 (0.94–2.15)	0.32 (0.19–0.51)	< 0.001
POD7	0.73 (0.46–0.99)	0.21 (0.14–0.35)	< 0.001
CRP (mg/L)			
POD1	81.00 (46.80–121.53)	40.44 (21.87–71.72)	0.045
POD2	247.89 (219.44–257.00)	174.57 (130.02–237.79)	< 0.001
POD3	201.72 (141.88–261.45)	179.50 (138.12–246.40)	0.081
POD5	107.16 (70.97–186.72)	89.52 (52.45–129.37)	0.007
POD7	80.70 (73.40–193.69)	51.85 (29.25–116.08)	0.001
DFA (IU/L)			
POD3	789.75 (67.75–3906.50)	32.00 (30.00–2802.90)	0.004

*CRP* C-reactive protein, *WBC* white blood cell count, *PCT* procalcitonin, *POD* post-operative day, *CRPF* clinically relevant pancreatic fistula, *LPD* laparoscopic pancreaticoduodenectomy, *IQR* interquartile range, *DFA* drain fluid amylase

### ROC analysis

**Fig. 2:**
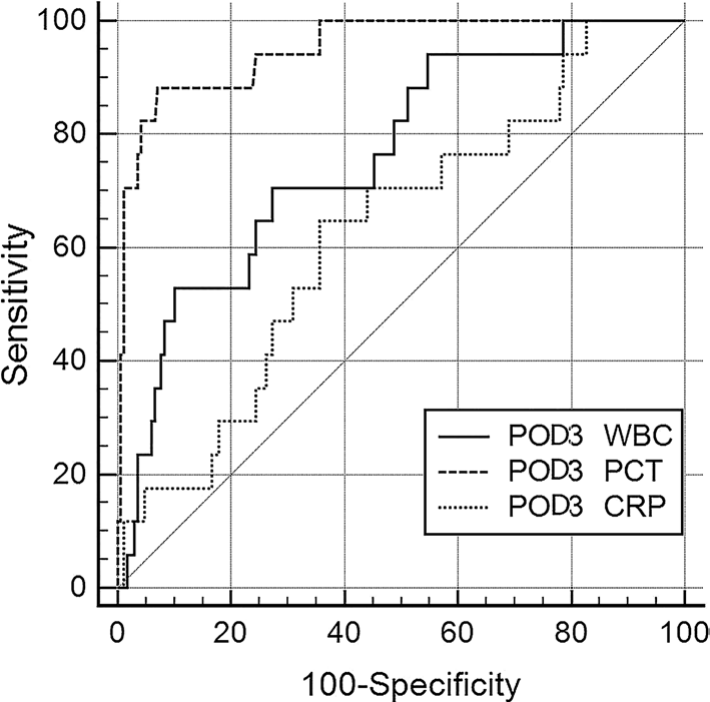
ROC curves for WBC counts, PCT levels, and CRP levels on POD 3 for predicting CRPF occurrence after LPD. The AUCs of the ROC plots for WBC, PCT levels, and CRP levels were 0.762, 0.951, and 0.629, respectively. PCT = 2.10 ng/mL had a sensitivity of 88.2% and specificity of 92.9%; CRP = 201.4 mg/L had a sensitivity of 64.7% and specificity of 64.3%; WBC = 14.7 × 10^9^/L had a sensitivity of 70.6% and specificity of 72.6%. There were significant differences between the ROC analyses (*P* < 0.05, Delong’s test). *ROC* receiver operating characteristic, *CRP* C-reactive protein, *WBC* white blood count, *PCT* procalcitonin, *POD* post-operative day, *LPD* laparoscopic pancreaticoduodenectomy

The AUC and cut-off values of WBC, PCT, CRP, and POD3 DFA levels were determined using ROC analysis and are listed in Table 3. Based on the AUCs obtained from the ROC plots, the diagnostic accuracy of WBC, CRP, and POD3 DFA levels were ‘fair’ (AUC < 0.8), while the accuracy of PCT levels on PODs 2, 3, 5, and 7 was ‘excellent’ (AUC > 0.9). The AUC obtained from the ROC plot of PCT levels was significantly higher than that of WBC and CRP levels on PODs 1, 2, 3, 5, and 7 (*P* < 0.05, Delong’s test). The highest AUC value for PCT was achieved on POD 3 [PCT > 2.10 ng/ml (AUC 0.951; sensitivity 88.2%, specificity 92.9%, *P* < 0.001)] (Fig. 2).

**Table 3:** ROC analysis for the prediction of CRPF occurrence after LPD

Days	Variables	Cutoff	AUC (95%CI)	*P* value	Sensitivity	Specificity
POD1	WBC	12.44	0.576 (0.417–0.734)	0.317	68.8%	51.9%
	PCT	0.65	0.788^c^ (0.650–0.925)	< 0.001	81.3%	75.9%
	CRP	64.58	0.625 (0.467–0.783)	0.099	62.5%	68.4%
POD2	WBC	18.84	0.695^a^ (0.541–0.849)	0.010	62.5%	72.6%
	PCT	3.30	0.931^c^ (0.875–0.987)	< 0.001	81.3%	93.7%
	CRP	216.93	0.794 (0.711–0.876)	< 0.001	87.5%	71.5%
POD3	WBC	14.71	0.762^a,b^ (0.647–0.877)	< 0.001	70.6%	72.6%
	PCT	2.10	**0.951**^**c**^** (0.903–0.999)**	< 0.001	88.2%	92.9%
	CRP	201.40	0.629 (0.495–0.762)	0.081	64.7%	64.3%
	DFA	79.50	0.694 (0.555–0.833)	0.008	76.5%	70.2%
POD5	WBC	12.70	0.732^a^ (0.600–0.863)	0.002	56.3%	81.6%
	PCT	0.91	0.930^c^ (0.887–0.972)	< 0.001	93.8%	87.9%
	CRP	95.01	0.702 (0.572–0.832)	0.008	81.3%	60.3%
POD7	WBC	10.90	0.776 (0.632–0.919)	0.001	85.7%	60.9%
	PCT	0.455	0.905^c^ (0.844–0.966)	< 0.001	85.7%	86.2%
	CRP	73.20	0.746 (0.631–0.861)	0.059	85.7%	64.5%

There were significant differences in the AUC values obtained from the ROC curves for the ^a^WBC counts and PCT levels, the ^b^WBC counts and CRP levels, and the ^c^PCT and CRP levels (*P* ˂ 0.05, Delong’s test). Bold font indicates significance at the reported analysis

*ROC* receiver operating characteristic, *AUC* area under the curve, *CRP* C-reactive protein, *WBC* white blood cell, *PCT* procalcitonin, *POD* post-operative day, *CRPF* clinically relevant pancreatic fistula, *LPD* laparoscopic pancreaticoduodenectomy, *DFA* drain fluid amylase

### Multivariate analyses of predictive risk factors for CRPF in patients undergoing LPD

Multivariate logical regression analyses revealed that POD3 PCT levels (odds ratio (OR) = 3.303, 95% confidence interval (CI) [1.902–5.736], *P* < 0.001) and WBC counts (OR = 0.282, 95%CI [0.019–4.115], *P* = 0.004) were independent predictive factors for CRPF after LPD (Table 4).

**Table 4:** Multivariate analyses of predictive risk factors for CRPF in patients undergoing LPD

Variables	OR	95% Cl	*P* value
Blood loss (ml**)**	1.001	0.994–1.009	0.782
BMI (< 25 kg/m^2^ vs ≥ 25 kg/m^2^)	0.472	0.005–4.431	0.511
Pancreatic duct diameter (< 3 mm vs ≥ 3 mm)	0.842	0.157–4.534	0.843
Gland texture (Soft vs Firm)	0.282	0.019–4.115	0.355
POD3 WBC	1.269	1.081–1.490	0.004
POD3 CRP	0.996	0.982–1.009	0.555
POD3 PCT	3.303	1.902–5.736	< 0.001
POD3 DFA	1.000	0.999–1.000	0.807

*OR* odds ratio, *CI* confidence interval, *BMI* body mass index, *CRP* C-reactive protein, *WBC* white blood cell count, *PCT* procalcitonin, *POD* post-operative day, *CRPF* clinically relevant pancreatic fistula, *LPD* laparoscopic pancreaticoduodenectomy, *DFA* drain fluid amylase

## Discussion

Post-operative pancreatic fistula (POPF) is a life-threatening complication after LPD. Various methods have been reported to predict and prevent POPF after OPD. However, LPD is a technically more demanding procedure that is only performed at a select number of surgical centers. As such, there are limited reports on the prediction of POPF after LPD. The current study was conducted at a high-volume center with nearly 200 cases of LPD being performed per year. The results of this study showed that WBC, CRP, and PCT levels were higher among patients with CRPF compared to those without CRPF. WBC counts are affected by factors other than inflammation and infection, such as trauma, acute blood loss, and medications, making this a less reliable marker than CRP and PCT levels for predicting CRPF.

CRP is the first acute reactant synthesized in the liver, with a half-life of 19 h. CRP levels rise above normal values within 6 h and peak at 48 h after stimulation [23]. Serum CRP levels are determined by the rate of synthesis. In the absence of additional inflammatory stimuli, CRP levels gradually decline after surgery on PODs 2 and 3. However, in the presence of post-operative complications, such as POPF, CRP levels will continued to rise [24]. In this study, post-operative CRP levels in the CRPF group peaked on POD 2, while levels in the non-CRPF group peaked on POD 3. This suggests acute activation of inflammatory stimuli in patients with CRPF after LPD. POPF and elevated CRP levels have been found to be associated with post-operative complications after various abdominal surgeries, including pancreatic [25] and colorectal surgeries [26]. In recent years, several studies have demonstrated that CRP levels can predict POPF development after OPD [27–29]. Kanda et al. [24] reported that a steep rise in serum CRP levels in the early post-operative period was predictive of CRPF after OPD. However, the AUC of the ROC plots for the Δ (POD3-POD1) CRP level was only 0.767, and the diagnostic accuracy of PODs 1, 3, and 5 CRP levels was lower (AUC 0.534–0.684). Malya et al. [27] reported that CRP levels > 19 mg/dL on POD 5 were predictive of CRPF after OPD with a high AUC value (0.851). However, the number of patients with grade C fistula was significantly higher in the study by Malya et al. compared to the current study, which may have led to different results. Guilbaud et al. [28] showed that a serum CRP level ≥ 100 mg/L on POD 1 was an independent predictor of POPF after OPD. However, the authors included grade A, B, and C POPF in their study. According to the 2016 ISGPS criteria, biochemical leak (grade A fistula) is no longer regarded as a true POPF, and research on CRPF may be more clinically meaningful. It should also be noted that none of the previous studies measured other inflammatory markers.

PCT is considered to be a marker of severe bacterial infections and has the potential to distinguish between infectious and non-infectious systemic inflammation [30]. PCT can also predict anastomotic leakage after colorectal surgery [31, 32]. However, PCT has not been extensively used as an early marker of complications after pancreatic surgery. To date, only a few studies have discussed the role of PCT for predicting complications after OPD. Bianchi et al. [33] showed that PCT on POD 2 was the best predictor of infectious complications after OPD. Another study found that pre-operative PCT levels were superior to pre-operative CRP levels for predicting infectious complications after OPD [17]. Giardino et al. [15] demonstrated that PCT > 0.4 mg/dl on POD 1 could be an early predictor of CRPF after OPD. Similarly, in the current study, we found that PCT levels were a better predictive marker of CRPF development after LPD compared to CRP levels. PCT is a specific marker for bacterial infections and may not best reflect the inflammatory status (including chemical inflammation caused by pancreatic fistula) unlike CRP [27]. Thus, we hypothesize that POPF following LPD is initiated by biochemical leakage (BL) without signs of infection. However, persistent BL may result in CRPF, which is closely associated with bacterial infection around the anastomotic site [34]. Some studies have classified POPFs as organ-space surgical site infections [18]. Additionally, the inflammatory response following laparoscopic surgery and open surgery differ. Most reported trials showed that CRP peak levels were significantly higher for open cholecystectomy than for laparoscopic cholecystectomy [35, 36]. Similarly, Schwenk et al. [37] reported that CRP levels were lower after laparoscopic surgery than open colorectal resections. This suggests that laparoscopic surgery may weaken the inflammatory response and reduce CRP levels. Several comparison studies between LPD and OPD suggest that LPD is associated with a lower estimated intra-operative blood loss and tissue ischemia, which may further weaken the inflammatory response [38, 39]. We suspect that with the weakening of the inflammatory response, other factors such as infection that cause CRP to rise may become prominent; infection can increase CRP and PCT levels after pancreatectomy [17]. In summary, the good performance of PCT for predicting CRPF after LPD may be related to the weaker inflammatory response following laparoscopic surgery and inevitable concurrent infections.

Drain fluid amylase content is superior for determining the presence of POPF because it directly reflects the leakage of pancreatic fluids. However, amylase concentration can be strongly influenced by the amount of exudative ascites fluid and the efficacy of drainage [24], indicating that it does not always increase parallel to the exacerbation of POPF. According to the 2016 ISGPS criteria, grade B fistula is diagnosed when there is a clinically apparent symptomatic fistula with persistent drainage > 3 weeks. Therefore, the diagnosis of CRPF by DFA usually lags behind. In our study, POD3 DFA was of low diagnostic value (AUC 0.694) in the diagnosis of CRPF. This further reflects the importance of inflammatory indicators in early prediction of CRPF. A soft pancreas and small pancreatic duct have been widely reported as risk factors for POPF [12, 40]. However, when they were combined with inflammatory indicators in multivariate analysis, we found that inflammatory indicators become the predictive risk factors for CRPF. The results of the present study suggest that POD3 PCT could be an important marker used to tailor the post-operative management of LPD patients. When a patient's POD3 PCT is greater than 2.1 ng/ml after LPD, several preventive measures including early imaging techniques, evaluation of antibiotic treatment, and percutaneous drainage in the presence of intra-abdominal collections may be considered.

The present study has some limitations, including small sample size and the retrospective nature of the study. Future larger prospective studies are required to assess the validity and reliability of the present data. In the future, we plan to design prospective controlled trials to compare the differences in post-operative inflammatory response between LPD and OPD and to assess the ability of inflammatory markers to predict other infectious complications after LPD.

## Conclusions

Both CRP and PCT levels can be used to predict CRPF development after LPD. PCT levels > 2.10 ng/ml on POD 3 after LPD are superior predictive markers of CRPF compared to CRP levels. Early recognition of CRPF after LPD using these parameters can help surgeons to intervene in the early stages and reduce post-operative morbidity and mortality.

## Data Availability

The datasets generated and analyzed during the present study are available from the corresponding author on reasonable request.

## Abbreviations

PCT: Procalcitonin
CRP: C-reactive protein
WBC: White blood cell
LPD: Laparoscopic pancreaticoduodenectomy
OPD: Open pancreaticoduodenectomy
CRPF: Clinically relevant pancreatic fistula
POPF: Post-operative pancreatic fistula
ROC: Receiver operating characteristic
AUC: Area under the curve
POD: Post-operative day
CI: Confidence interval
IQR: Interquartile range
OR: Odds ratio
DFA: Drain fluid amylase
